# A polarization image enhancement method for glioma

**DOI:** 10.3389/fnins.2023.1163701

**Published:** 2023-07-13

**Authors:** Yi-Rong Liu, Chao-Feng Liang, Han-Qiao Zhao, Yun-Mou Ou, Jian Wu

**Affiliations:** ^1^School of Medicine, Tsinghua University, Beijing, China; ^2^Tsinghua Shenzhen International Graduate School, Tsinghua University, Shenzhen, China; ^3^Department of Neurosurgery, The Third Affiliated Hospital of Sun Yat-sen University, Guangzhou, China

**Keywords:** glioma tissue, polarization imaging, image enhancement method, Mueller matrix elements, image evaluation

## Abstract

Polarization imaging technique (PIT) based on a backward scattering 3 × 3 Mueller matrix polarization imaging experimental setup is able to study the optical information and microstructure of glioma and non-glioblastoma tissues from clinical treatment. However, the image contrast of Mueller Matrix Elements (MME) is far from sufficient to provide supplemental information in the clinic, especially in off-diagonal MME. The aim of this work is to propose an innovative method to improve the contrast and quality of PIT images of glioma and non-glioma tissues. The work first confirms the robustness of the method by evaluating the enhanced images and assessment coefficients on *ex vivo* unstained glioma and non-glioma sample bulks, then the optimal enhancement results are tested and presented based on the multi-sample tests. This PIT image enhancement method can greatly improve the contrast and detailed texture information of MMEs images, which can provide more useful clinical information, and further be used to identify glioma and residues in the intraoperative environment with PIT.

## 1. Introduction

As a non-contact and *in situ* technique, PIT has many unique advantages that can provide different and complementary microstructural and optical information of a sample compared to an intensity-based imaging method (Alali and Vitkin, 2015; Chandel et al., 2016; Dong et al., 2016; He et al., 2017), the PIT backscatter method and the following analysis can distinguish cancer tissue from healthy tissue (Menzel et al., 2019; Schucht et al., 2020; Rodríguez-Núñez and Novikova, 2022), and there is an emerging interest in the applications of PIT for biomedical tissues, where the polarized light typically suffers multiple scatterings before being eventually detected (Alali and Vitkin, 2015; He et al., 2017). Since the Mueller matrix provides a characterization of the polarization properties and contains abundant microstructural and optical information of the sample (Pezzaniti and Chipman, 1995; Chung et al., 2002), PIT is becoming increasingly attractive for differentiating pathological structural features of different tumor types (Du et al., 2014; Wang et al., 2014; Menzel et al., 2019; Schucht et al., 2020; Rodríguez-Núñez and Novikova, 2022). It is based on the analysis of the modification of the polarization state of incident polarized light due to the interaction with the sample to be examined, which can be described by the Mueller matrix. In our previous work, it was confirmed that PIT can investigate optical information and microstructures of glioma and non-glioma tissues from clinical treatment based on backward scattering 3 × 3 Mueller matrix polarization imaging experimental setup, and the polarization properties of the glioma and non-glioma brain tissues have been characterized and reported (Liu et al., 2022).

However, the experiment results showed that the glioma brain tissues have larger magnitudes of diagonal Mueller matrix elements and better element image contrast, while the off-diagonal Mueller matrix elements have smaller magnitudes and lower image contrast of the elements. In addition, the hypervascular glioma is always in close proximity to blood, water, tissue fluids, and other various complex components resulting from surgical resection during the operation, and these fluids and components can partially change the absorption and scattering of the polarized light, and have an effect on the Mueller matrix of the clinical and surgical samples. Therefore, even though their microstructures are different, these complex components can ultimately account for a similar optical PIT response, increasing the error in the subsequent calculation of PIT parameters. Therefore, there is an urgent need to develop a PIT image enhancement method for glioma and non-glioma tissues. In this paper, a PIT image enhancement method based on Central Moment Coefficients (CMCs) is proposed to improve the characterization of glioma and non-glioma brain tissues from the clinics, especially the off-diagonal Mueller matrix elements images.

This article describes the principle of the proposed PIT enhancement method and briefly introduces the method for preparing clinical specimens of surgical glioma for testing. First, the original and enhanced PIT images are presented, and assessment coefficients are used to validate the proposed enhancement method solid. Second, this article also suggested and tested two important factors affecting the image enhancement effect, reporting optimal results based on multi-sample tests. Then the inadequacy and future work of this research is discussed and the last section concludes the article.

## 2. Methods and materials

### 2.1. Tested PIT images

Twenty unstained thick bulk glioma samples (including glioma and non-glioma brain tissue, which contained no glioma cells) from the operation are involved to test the availability of the enhancement method proposed in this article, provided by the Department of Neurosurgery, General Hospital, Tianjin Medical University. The use of the glioma clinical samples in this study includes both glioma and non-glioma regions, and the preprocessing and enhancing process is performed in each region independently.

Since distinguishing the glioma region from the non-glioma region is the priority, the PIT images of the samples are then captured by the portable 3 × 3 Mueller matrix backward scattering configuration to obtain the PIT images and parameters, which indicated less but enough polarization information for polarization measurement of clinical glioma tissue bulks. Without the circular polarizations in the construction of 3 × 3 Mueller matrix measurement, it significantly simplifies the experimental geometry, which is particularly appropriate for clinical samples (Forward et al., 2017; Khaliq et al., 2021). The parallel source (630 nm, BT-TCL24, BTOS Telecentric Optical, China) provides a circular illumination area of 60 millimeters in diameter with a central wavelength of 630 nm. The polarization states of the incident light are generated by a polarization state generator (PSG) including a polarizer (P1, Thorlabs, United States). The polarized light backscattered from the sample on stage then passes through the polarization state analyzer (PSA) with a polarizer (P2, Thorlabs, United States) before being detected by a CCD monochrome industry camera (MER-503-36U3M/C, Daheng Imaging, China) to capture the resulting PIT images. PSG and PSA are designed as compact modules. During the experiments, P1 and P2 rotate 0°, 45°, 90°, and 135° to generate different PSG and PSA states driven by two DC servo motors (MR-J3-40A, Mitsubishi Electric, China) which are covered by driven gears. Due to careful calibration, the maximum errors of the absolute values of all elements of the Mueller matrix are less than 0.04.

### 2.2. Preprocessing of PIT images

The acquired PIT data is used to calculate the Mueller matrix of the detected sample, which represents the transfer function of the sample in its interactions with polarized light, and the MMEs are associated with specific biological or clinical properties (Pezzaniti and Chipman, 1995).

To perform the PIT image enhancement procedure, the frequency distribution histograms (FDHs) and central moment coefficients (CMCs) P3 and P4 of two-dimensional MME images (He et al., 2015) are calculated to separate the most dominant features of the microstructure:


(1) $$P3=\frac{E{\left(X-\mu \right)}^{3}}{\sigma^{3}}$$

(2) $$P4=\frac{E{\left(X-\mu \right)}^{4}}{\sigma^{4}}$$

Where X is the variable of MME, and P3 and P4 refer to the skewness and kurtosis of X (Grimmett, 2001; Ushenko et al., 2014), respectively. Specifically, P3 represents the degree of asymmetry of the FDH curve, if P3 > 0, it means that the end of the FDH curve on the right is longer than that on the left, while P3 < 0 indicates the end of the FDH curve left longer than right. P4 represents the kurtosis of the FDH curve, indicating the sharpness of the peak of the FDH distribution.

In this paper, the backscattering 3 × 3 Mueller matrix of glioma and non-glioma regions is first calculated, and then the FDHs and the corresponding CMCs P3 and P4 of each MME are calculated. Based on the P3 and P4 values calculated from the glioma and non-glioma samples, the following image enhancement process can be performed.

### 2.3. PIT image enhancement method

As we know, values of diagonal MMEs are closely related to the depolarization capability of the samples. Due to the analysis of the experiment results, it can be observed that the non-glioma tissue has smaller diagonal m22 and m33 element values than glioma tissue, which shows that there is a stronger depolarization power for non-glioma tissue than glioma tissue. This characterization can be attributed to the changes in cell density during the formation and development of glioma tissue (Qi et al., 2013). Therefore, the enhancement of images of MMEs is essential.

**Figure 1:**
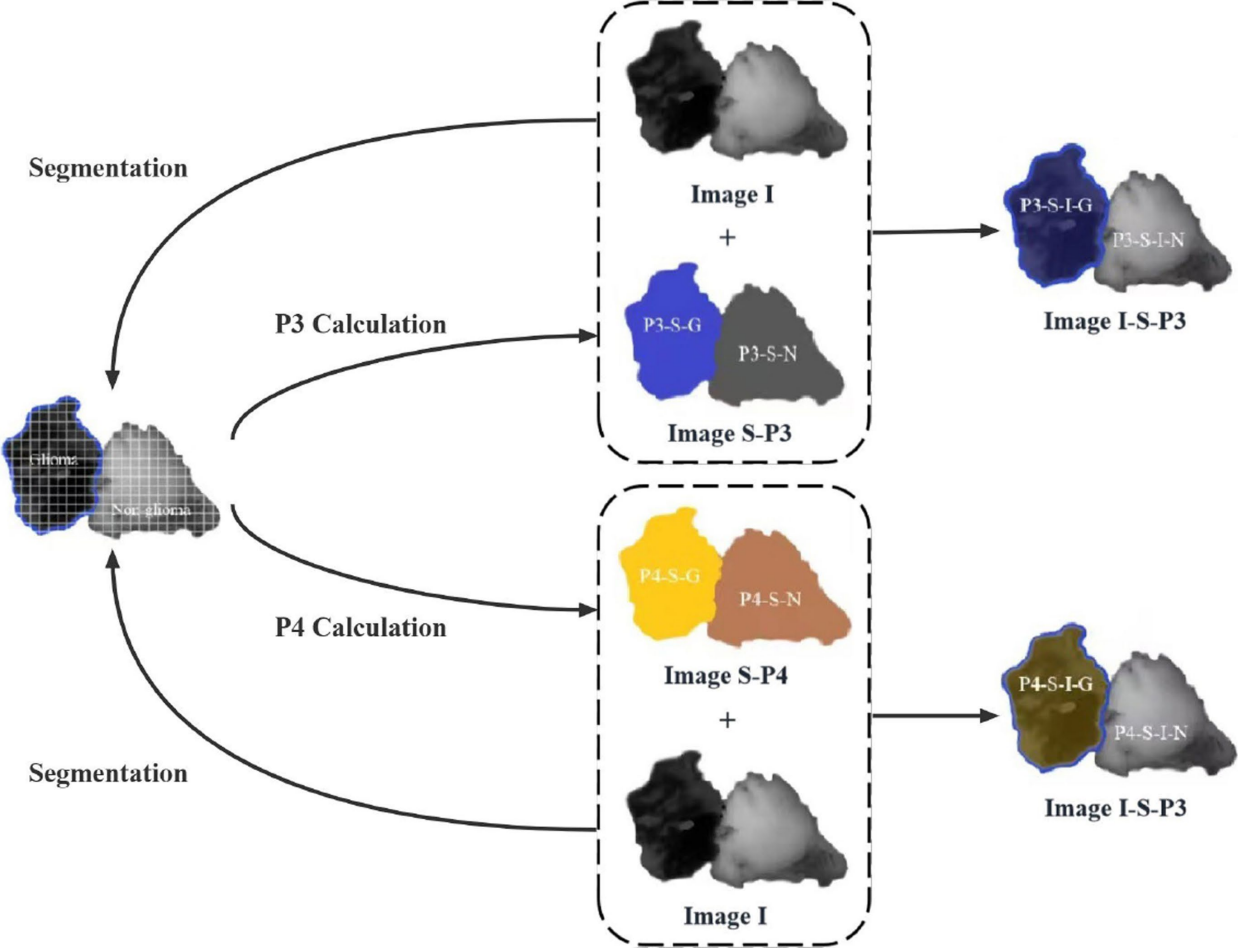
The schematic of the principle of PIT image enhancement method with enhancing-P3 and enhancing-P4 matrices.

Once the MMEs-PIT images are obtained and P3 and P4 are calculated, the image enhancement method can be applied. The PIT image contains both a glioma and a non-glioma region, and the P3 and P4 values are different in the PIT image. The principle of the PIT image enhancement method is based on the difference of P3 and P4 values, as shown in Figure 1: First, the glioma and non-glioma regions of the original PIT image I are segmented into a certain number of non-overlapping sub-regions, represented by as segmented image I-S, and P3 and P4 values of sub-regions are calculated accordingly to better characterize the local features and displayed in pseudocolors, which can show display the kurtosis and skewness information to compare differences between glioma and non-glioma region, constructing a P3 enhancement matrix and a P4 enhancement matrix (image S-P3 and image S-P4 in Figure 1).

They are then overlaid on the original image I to create the enhanced P3 and P4 images that contain not only the PIT information of the original image but also information about the local kurtosis and skewness of the glioma and non-glioma region of P3-enhancement matrix and P4- enhancement matrix, shown as P3 enhanced image and P4 enhanced image (image I-S-P3, image I-S-P3, image I-S-P4, and image I-S-P4 in Figure 1). the principle is as follows:


(3) $$I-S-P3{\left(i,j\right)}_{seg-N}=\alpha I\left(i,j\right)+\beta P3{\left(i,j\right)}_{seg-N}$$

(4) $$I-S-P4{\left(i,j\right)}_{seg-N}=\alpha I\left(\mathrm{i,}j\right)+\beta P4{\left(i,j\right)}_{seg-N}$$

In which α and β indicate the overlapping scale for the original image and P3, P4 enhancement matrix, and α + β = 1, while N indicates the segmentation mode of original images, and *N* = 2, 4, 10, 20, 25. They take into account two important factors affecting the enhancement effect of the method: (1) the overlapping scale between the original image and P3 and P4 enhancement matrices. (2) Original image segmentation mode. These factors are tested and discussed in the next section. For an original image of i × j pixels, N-segmentation mode refers to the original PIT images segmented into i × j /N^2^ sub-regions, and the P3 and P4 values of each sub-region are calculated. Thus, the enhancement of PIT images of Mueller matrix elements of glioma and non-glioma tissues is realized.

## 3. Results

### 3.1. Results of PIT images preprocessing

**Figure 2:**
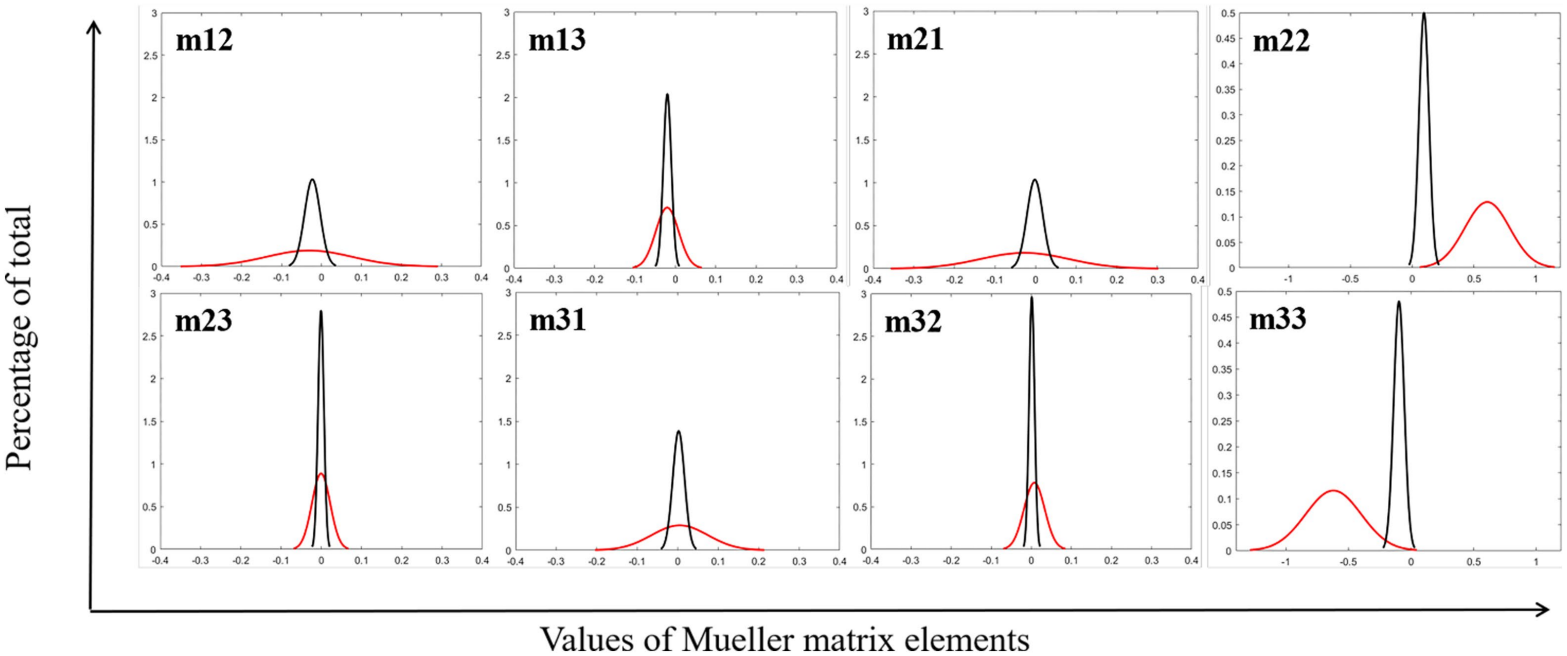
FDH distribution curves of MMEs of glioma (red lines) and non-glioma region (black lines).

First, the backscattered 3 × 3 Mueller matrices of the test samples are calculated, the elements of which are normalized by m11. Images of diagonal elements have high contrast between glioma and non-glioma regions, while off-diagonal elements have low contrast to distinguish them. Then the FDH distribution curves of glioma and non-glioma regions of the study samples are shown in Figure 2, and the respective P3 and P4 values were calculated and listed in Table 1.

**Table 1:** Values of parameter P3 and P4 of the MMEs for glioma and non-glioma regions.

	m12	m13	m21	m22	m23	m31	m32	m33
Glioma-P3	−0.114	0.008	−0.199	−0.469	−0.041	0.879	0.004	−0.263
Non-glioma-P3	−0.967	−1.684	−0.081	1.964	−0.098	0.129	0.151	−1.923
Glioma-P4	3.976	4.531	3.787	2.038	4.090	6.176	4.513	2.492
Non-glioma-P4	5.317	8.024	5.929	2.996	8.455	8.508	7.958	3.597

Figure 2 and Table 1 show that the fitted FDH distribution curves of MMEs of glioma and non-glioma regions are transformed into the quantitative CMCs: skewness P3, and kurtosis P4, which characterize the position and shape of the FDHs of the tested samples. It can be seen from Figure 2 and Table 1 that the FDHs and parameters P3 and P4 clearly and quantitatively reveal the main structural features of the tested samples, and glioma and non-glioma regions have significantly different values of parameters P3 and P4, characterized by different characteristics of FDH distribution curves, which are caused by differences in the optical microstructural features of glioma and non-glioma regions. Based on the differences between parameters P3 and P4 for each MME and the appropriate segmentation mode of original PIT images, the P3-enhancement matrix (image S-P3) and P4-enhancement matrix (image S-P4) are constructed, and then combined through the appropriate overlapping scale (α and β) with original images, the enhancement of PIT images is finally implemented.

### 3.2. Results of enhanced PIT images with parameter P3 and P4

This section presents the enhanced image results of the Mueller matrix elements of glioma and then assesses the original image and the enhanced image quality using the assessment parameters C and MG to show improved contrast and detailed texture information:


(5) $$C=\underset{\delta }{\sum }\delta_{DN}{\left(i,j\right)}^{2}p_{\delta }\left(i,j\right)$$

(6) $$\begin{array}{c} \mathrm{MG}=\frac{1}{\left(m-1\right)\left(n-1\right)}\\ \times \overset{m-1}{\underset{i=1}{\sum }}\overset{n-1}{\underset{j=1}{\sum }}\sqrt{\frac{{\left[DN\left(i,j\right)-DN\left(i+1,j\right)\right]}^{2}+{\left[DN\left(i,j\right)-DN\left(i,j+1\right)\right]}^{2}}{2}} \end{array}$$

Where δDN(i,j) represents the gray difference between adjacent pixels, and pδ(i,j) represents the pixel distribution probability when the gray difference between adjacent pixels is δ. In addition, C refers to the contrast of the tested images, which shows the clarity of the images, while MG refers to the mean gradient, a larger MG indicates that the tested PIT images are more sensitive to detailed texture information. And normalized assessment coefficients Cp and MGp are also calculated:


(7) $$\mathrm{Cp}=\frac{C\left(e\right)-C\left(o\right)}{C\left(o\right)}$$

(8) $$\mathrm{MGp}=\frac{MG\left(e\right)-MG\left(o\right)}{MG\left(o\right)}$$

Where C(e) and MG(e) represent the contrast and mean gradient values of the enhanced PIT images, while C(o) and MG(o) represent the contrast and mean gradient values of the original PIT images, respectively. They are normalized by the C and MG values of the original images to display the evaluation results. Therefore, assessment coefficients C, MG, Cp, and MGp are used to quantify the enhanced results, and both original and enhanced MMEs images are also presented to show the visual effect and differences directly.

### 3.3. Tests of overlay scale and evaluations of enhanced PIT images based on a single sample

This section shows the enhancement process and the results of enhanced PIT images with the enhancement method, using a single sample with diagonal and off-diagonal MME, on a fixed overlapping scale of *α* = 0.7, *β* = 0.3, and *N* = 10 (seg-10) of the original images for example.

**Figure 3:**
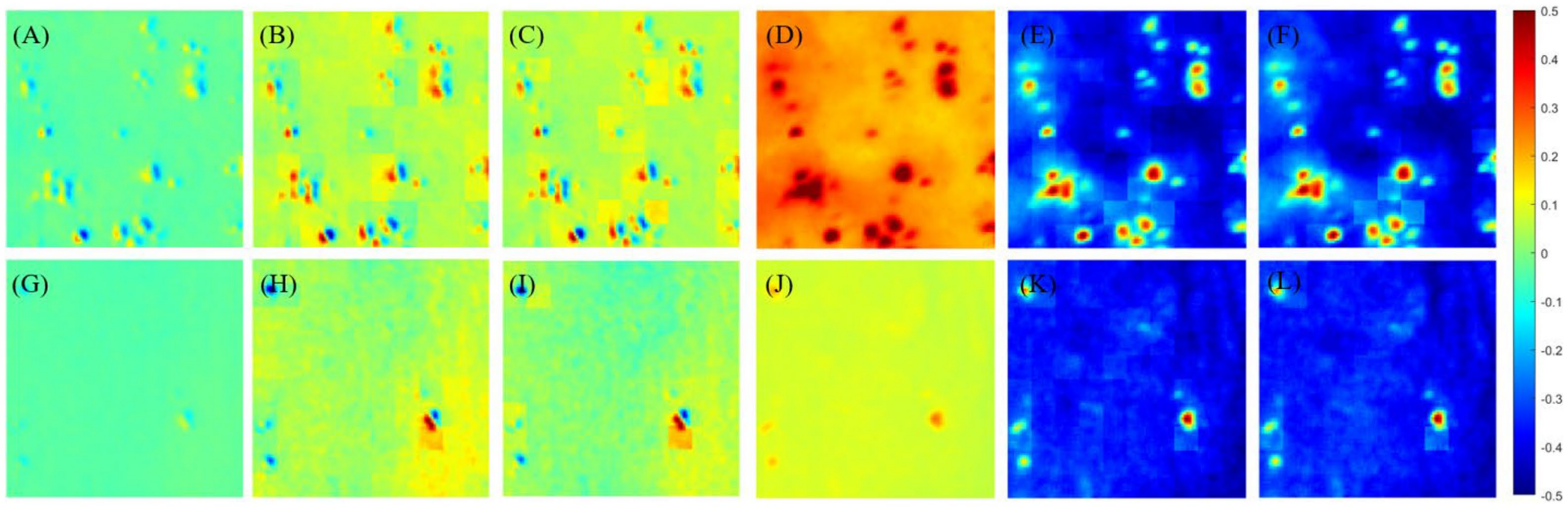
**(A)** Origin image I of glioma region of Mueller matrix element of m12, **(B)** P3-enhanced image I-S-P3 of **(A)**, **(C)** P4-enhanced image I-S-P4 of **(A)**, **(D)** origin image I of glioma region of Mueller matrix element of m22, **(E)** P3-enhanced image I-S-P3 of **(D)**, **(F)** P4-enhanced image I-S-P4 of **(D)**, **(G)** origin image I of non-glioma region of Mueller matrix element of m12, **(H)** P3-enhanced image I-S-P3 of **(G)**, **(I)** P4-enhanced image I-S-P4 of **(G)**, **(J)** origin image I of non-glioma region of Mueller matrix element of m22, **(K)** P3-enhanced image I-S-P3 of **(J)**, **(L)** P4-enhanced image I-S-P4 of **(J)**. The overlay scale of **(B,C,E,F,H,I,K,L)** are *α* = 0.7, *β* = 0.3.

The results are shown in Figure 3 and Table 2. Figure 3 shows the results of off-diagonal MME (using, e.g., m12) and diagonal MME (using, e.g., m22) with the proposed PIT image enhancement method. It shows the difference between the MMEs of glioma original image I (as shown in Figures 3A,D) and P3, P4-enhanced images I-S-P3, I-S-P4 (as shown in Figures 3B,C,E,F). Similarly, it also shows the differences between the non-glioma original image I (as shown in Figures 3G,J) and P3, P4-enhanced images I-S-P3, I-S-P4 (as shown in Figures 3H,I,K,L). During the enhancement process, the P3 and P4 values are calculated and form the P3 and P4-enhancement matrix (image S-P3, S-P4). It is easy to see that the P3 and P4 enhanced images I-S-P3 and I-S-P4 are greatly enhanced, and the contrast of the PIT image is improved, especially for the non-glioma region.

**Table 2:** The values of assessment coefficients C and MG of origin images I and enhanced images (I-S-P3, I-S-P4) with overlay scale *α* = 7, *β* = 3, and *N* = 10 of m12 and m22.

Assessment coefficients	Tested MME	Glioma region			Non-glioma region		
		I (*N* = 10)	I-S-P3	I-S-P4	I (*N* = 10)	I-S-P3	I-S-P4
C	m12	0.0012	0.0101	0.0109	0.0001	0.0055	0.0059
	m22	0.0011	0.0121	0.0117	0.0001	0.0074	0.0056
MG	m12	0.0131	0.0289	0.0266	0.0039	0.0227	0.0178
	m22	0.0148	0.0354	0.0291	0.0043	0.0275	0.0194

The assessment coefficients are then used to quantify the enhanced images of both diagonal and off-diagonal MMEs, and the results are presented in Table 2. It is clear that the assessment coefficients C and the MG are promoted for both glioma and non-glioma regions, especially non-glioma regions.

Although diagonal MME achieves a similar improvement in assessment coefficients C and MG, the enhanced PIT images do not receive a significant improvement. This may be because the original diagonal MMEs images are already high in contrast, so the enhanced images are less enhanced. Therefore, the proposed enhancement method is particularly useful for off-diagonal MMEs, and the results presented below are based on off-diagonal MMEs testing only.

### 3.4. Tests of optimal overlay scale and evaluations of enhanced PIT images based on multi-samples

**Figure 4:**
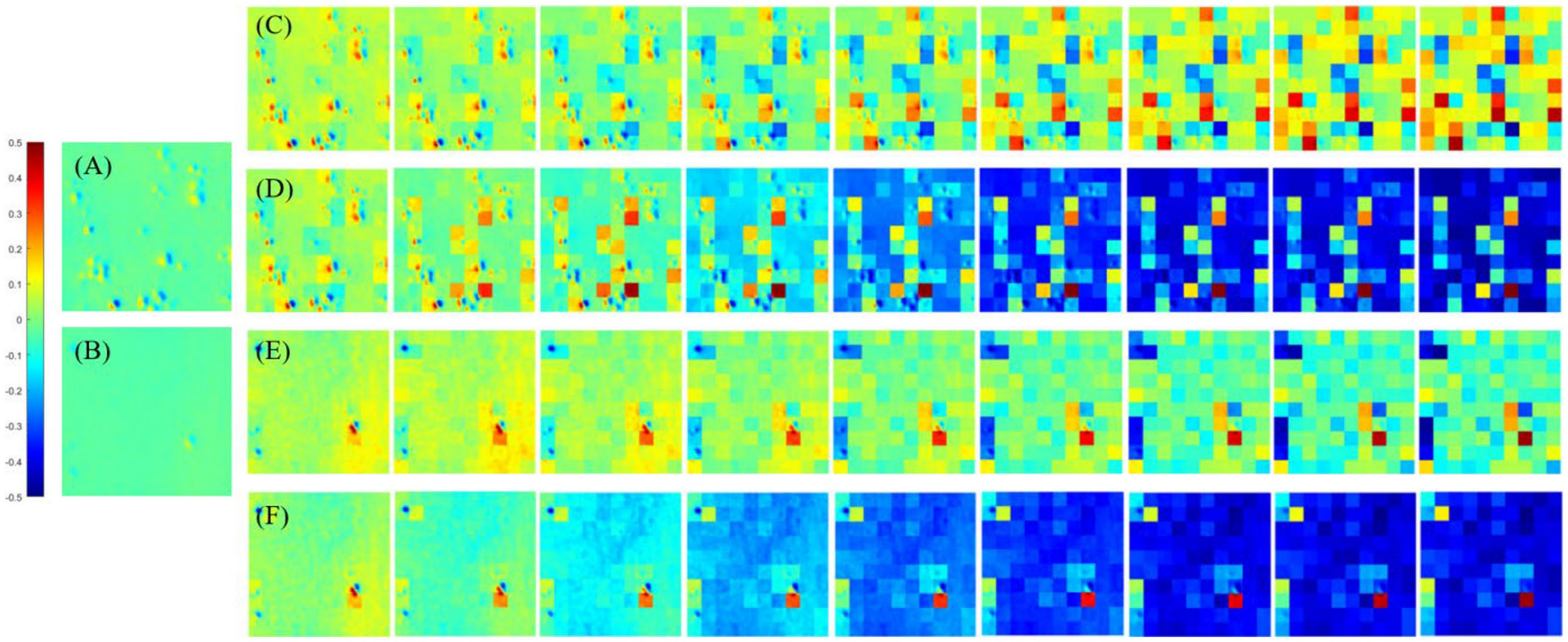
The origin m12 image of MMEs and P3 and P4 enhanced images with all overlay scales. **(A)** Glioma region of origin m12 image, **(B)** non-glioma region origin m12 image. **(C)** P3 enhanced images of glioma regions with overlay scales of *α* = 0.9, 0.8, …, 0.1 and *β* = 0.1, 0.2, …, 0.9. **(D)** P4 enhanced images of glioma regions with overlay scales of *α* = 0.9, 0.8, …, 0.1 and *β* = 0.1, 0.2, …, 0.9. **(E)** P3 enhanced images of non-glioma regions with overlay scales of *α* = 0.9, 0.8, …, 0.1 and *β* = 0.1, 0.2, …, 0.9. **(F)** P4 enhanced images of non-glioma regions with overlay scales of *α* = 0.9, 0.8, …, 0.1 and *β* = 0.1, 0.2, …, 0.9.

In this section, the PIT enhancement method is applied to the off-diagonal MMEs of all tested samples (both glioma and non-glioma regions), and the optimal overlapping scale of the original images and enhancement matrices is tested and determined. In particular, different overlapping scales from *α* = 0.1 to *α* = 0.9 and from *β* = 0.9 to *β* = 0.1 among the original images (*N* = 10) of MMEs are examined in both glioma and non-glioma regions. The original images of MMEs are shown in Figures 4A,B, while P3 and P4 enhanced images with different overlapping scales are shown in Figures 4C–F, using MME m12 as an example.

**Figure 5:**
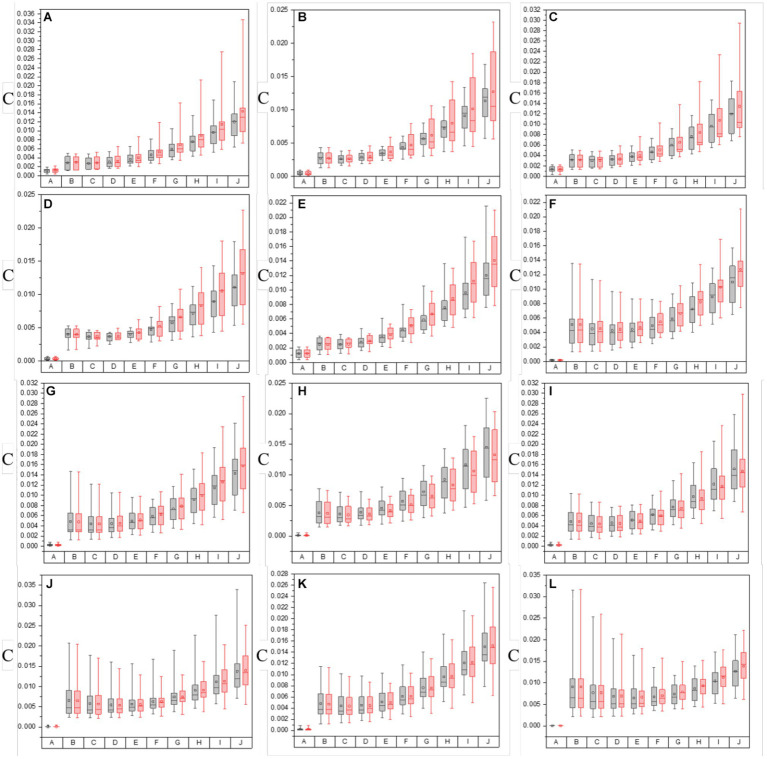
The values of C of both in the origin image group and groups with varying overlay scales between origin image and enhanced matrices of off-diagonal MMEs. **(A–F)** m12, m13, m21, m23, m31, and m32 for glioma regions, **(G–L)** m12, m13, m21, m23, m31, and m32 for non-glioma regions.

**Figure 6:**
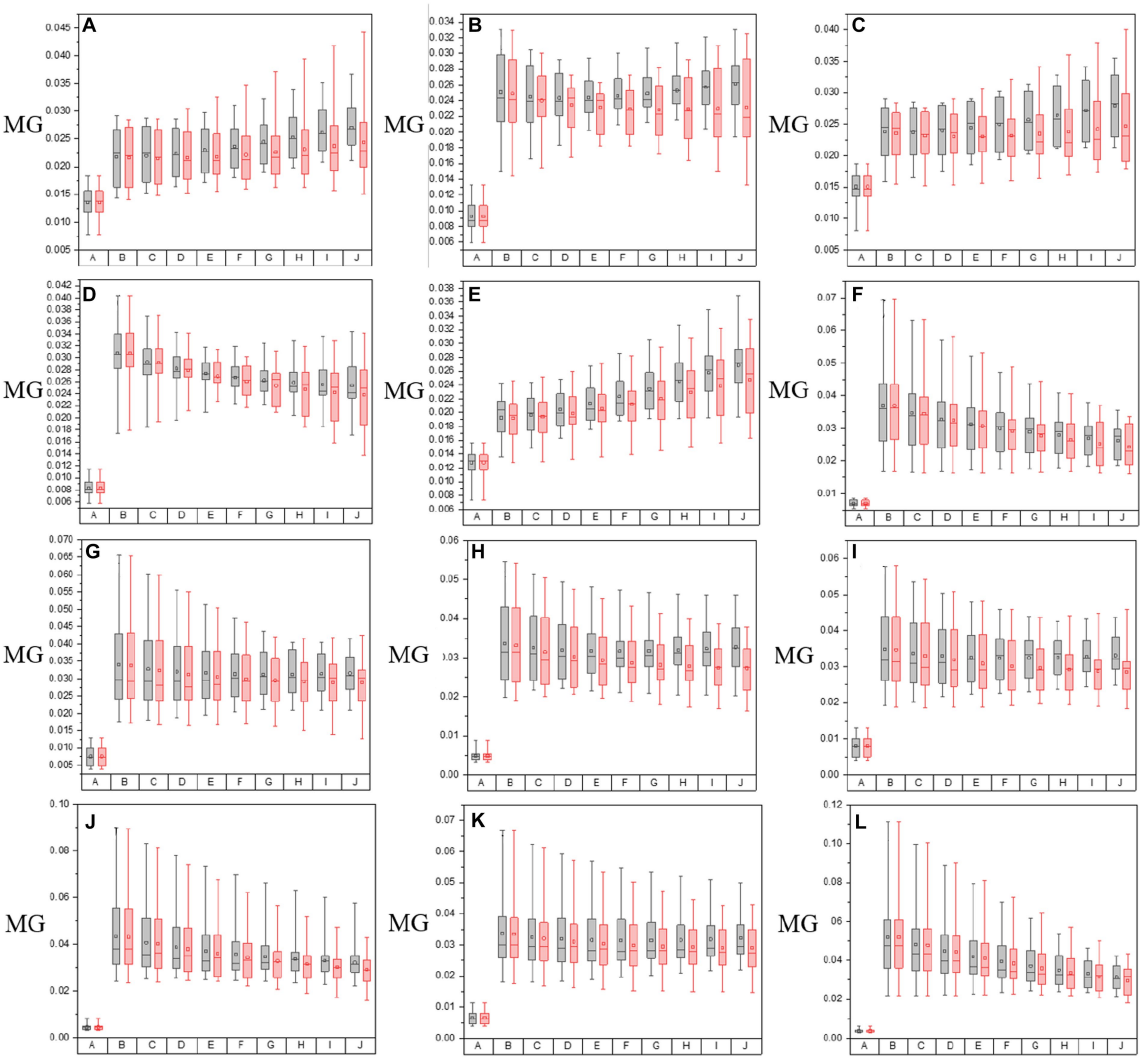
The values of MG of both in the origin image group and groups with varying overlay scales between origin image and enhanced matrices of off-diagonal MMEs. **(A–F)** m12, m13, m21, m23, m31, and m32 for glioma regions, **(G–L)** m12, m13, m21, m23, m31, and m32 for non-glioma regions.

Firstly, it can be obviously observed that the visual effect of enhanced PIT images has been significantly improved with the proposed method, based on the results presented in Figure 4, in which Figures 4A,B show the glioma and non-glioma region of origin image, and Figures 4C–F show the P3 and P4 enhanced images. However, it is clear that the enhanced images with overlay scale *α* = 0.9, 0.8 and 0.7, *β* = 0.1, 0.2 and 0.3 between the origin image and enhancing matrices are more acceptable and satisfactory, shown in Figures 4C–F. In addition, it is vivid that among all different overlay scales, *α* = 0.9 and *β* = 0.1 is supposed to be the optimal one, both for P3 and P4 enhancement, glioma regions and non-glioma regions, shown in Figures 4C–F, for it is with better PIT images visual effect and relatively large C and MG compared with the enhanced images with other overlay scales. Especially, the value of C of the glioma region and non-glioma region for P3 enhancement is 0.0160 and 0.0089, while for P4 enhancement is 0.0175 and 0.0096, respectively. The value of MG of the glioma region and non-glioma region for P3 enhancement is 0.0317 and 0.0249, while for P4 enhancement is 0.0289 and 0.0186, respectively. Then the same enhancing processes are performed on other off-diagonal MMEs, and the enhanced images are evaluated with assessment coefficients C and MG in both glioma and non-glioma regions, based on multi-sampled tests, and the results are shown in Figures 5, 6, in which the gray bar represents the results of P3-enhanced images, while the red bar represents the results of P4-enhanced images. What’s more, Figure 5 shows the evaluation results of coefficient C calculated from all tested samples of MMEs of glioma and non-glioma regions, while Figure 6 shows the evaluation results of coefficient MG. In each figure, group A represents origin images, while group B ~ group J represent enhanced images with overlay scale of *α* = 0.1, 0.2, …, 0.9 and *β* = 0.9, 0.8, …, 0.1.

Based on the results of these figures, when it comes to the study of the optimal overlay scale between origin images and P3 and P4 enhancing matrices, it can be seen that the values of assessment coefficients C and MG of P3 and P4 enhanced images are larger than those of origin images. Especially, the values of C and MG of non-glioma regions are larger than those of glioma regions, from the perspective of the evaluation of C and MG of MMEs of multi-sampled tests. Besides, For the evaluation of C, it shows that the values gradually increase with the overlay scale changing from *α* = 0.1, *β* = 0.9 to *α* = 0.9, *β* = 0.1, and the optimal overlay scale between origin and enhancing matrices is *α* = 0.9, *β* = 0.1, which is J group in Figure 5. In addition, compared with P3 enhanced images, P4 enhanced images gained better results. However, for the evaluation of MG, the values of which are all promoted for the enhanced images compared with origin images, while the values of MG present no dominant variation tendency with the overlay scale changing, so it is quite difficult to give definite optimal overlay scale that which group gained best result based on MG evaluation, and compared with P4 enhanced images, P3 enhanced images gained better results, which is different from the assessment of C.

To summarize, as for the tests for optimal overlay scale, the larger the proportion of the enhancing matrices S-P3 and S-P4 (larger β), the more prominent the effect of the pixelation, contributing to the larger values of the assessment coefficient MG. However, it may cause misleading information and increase the possibility of a shift of glioma or residual glioma when it is applied to the PIT images, which adversely affects the characterization of the original PIT images. On the contrary, the smaller the proportion of the enhancing matrices S-P3 and S-P4 (larger α), the slighter prominent the effect of pixelation, but the enhancing matrices S-P3 and S-P4 cannot be infinitesimal, otherwise, the information of P3 and P4 cannot be effectively used, which lead to weak enhancement effect, either. Therefore, it is necessary to conclude that the J group is the most appropriate and practical one in this research, in which the overlay scale is *α* = 0.9 and *β* = 0.1, based on the results of PIT images of off-diagonal MMEs and the evaluation coefficients, which is the enhancement effect when the overlay scale is optimal in this research. However, the other overlay scales of the enhancing matrices remain to be tested to further optimize the enhancement results.

### 3.5. Tests of segmentation mode and evaluations of enhanced PIT images based on a single sample

**Figure 7:**
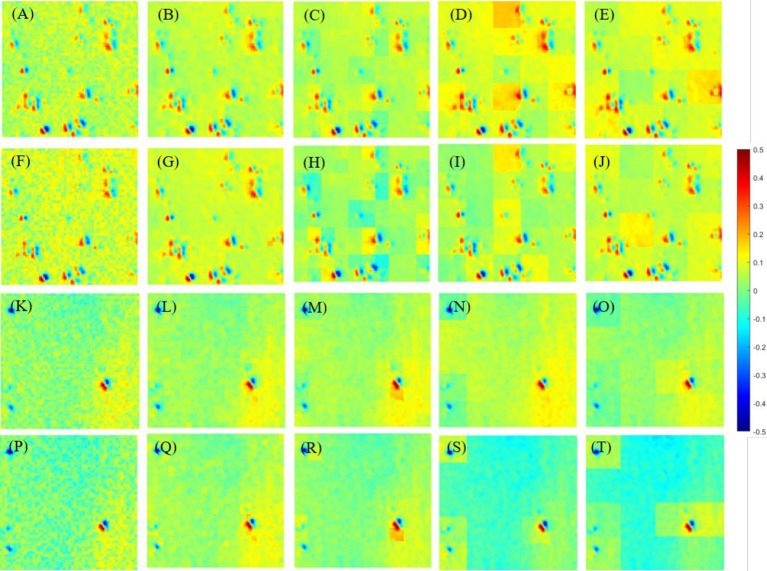
Images I-S-P3 and I-S-P4 with all segmentation modes. **(A–E)** Images I-S-P3 of glioma region with N=2, 4, 10, 20 and 25. **(F–J)** Images I-S-P4 of glioma region with *N* =2, 4, 10, 20 and 25. **(K–O)** Images I-S-P3 of non-glioma region with *N* =2, 4, 10, 20 and 25. **(P–T)** Images I-S-P3 of non-glioma region with *N* =2, 4, 10, 20 and 25.

In this section, the segmentation mode of the original PIT images is tested and determined under the condition that the overlapping scale is *α* = 0.9 and *β* = 0.1. Specifically, different segmentation modes (*N* = 2, 4, 10, 20, and 25) will be tested in off-diagonal MMEs in both glioma and non-glioma regions. The results are shown in Figure 7 and Table 3 using MME m12 as an example. The P3 and P4 enhanced PIT images with different segmentation modes for glioma and non-glioma regions are shown in Figure 7. For a quantitative comparison, the assessment coefficients C and MG values of original images and enhanced images with different segmentation modes are listed in Table 3.

**Table 3:** The values of assessment coefficients of origin image I and enhanced images with different segmentation modes of m12.

Assessment coefficients		Glioma region						……
		I	*N* = 2	*N* = 4	*N* = 10	*N* = 20	*N* = 25	……
C	I-S-P3	0.0012	0.0054	0.0029	0.016	0.0028	0.0027	……
	I-S-P4		0.0071	0.003	0.003	0.0029	0.0027	……
MG	I-S-P3	0.0131	0.0382	0.0224	0.0175	0.0213	0.0208	……
	I-S-P4		0.045	0.0216	0.0287	0.0223	0.0208	……

……	Non-glioma region					
……	I	*N* = 2	*N* = 4	*N* = 10	*N* = 20	*N* = 25
……	0	0.004	0.0014	0.0012	0.0012	0.0013
……		0.0056	0.0015	0.0012	0.0011	0.0013
……	0.0039	0.0357	0.0191	0.0176	0.0175	0.0174
……		0.0416	0.0193	0.0171	0.0174	0.0082

It is easy to see that the contrast of the enhanced images is favored over the contrast of the original image I shown in Figure 7, and the differences between them are quite clear. Briefly, different segmentation modes result in different display effects on PIT images. Among these segmentation modes, *N* = 4 and *N* = 10 performed best, for *N* = 2, 20, 25, they show different degrees of pixelation with strong edges in each sub-region.

Then, Table 3 quantifies the assessment coefficients results of enhanced images with different segmentation modes of glioma and non-glioma regions, it can be seen that assessment coefficients are promoted for all tested segmentation modes, especially for non-glioma regions. For the enhanced images, it gained better results when *N* = 2, both C and MG were promoted more than other segmentation modes, and the results of *N* = 4 and *N* = 10 are also acceptable and satisfactory. In combination with the results from Figure 7, it is reasonable to indicate that *N* = 4 and *N* = 10 is a good compromise.

### 3.6. Tests of optimal segmentation mode and evaluations of enhanced PIT images based on multi-samples

This section presents the results of enhanced PIT images of off-diagonal MMEs, including glioma and non-glioma regions, using the proposed enhancement method with different testing segmentation modes (*N* = 2, 4, 10, 20, and 25) and determined the overlapping scale of *α* = 0.9 and *β* = 0.1.

**Figure 8:**
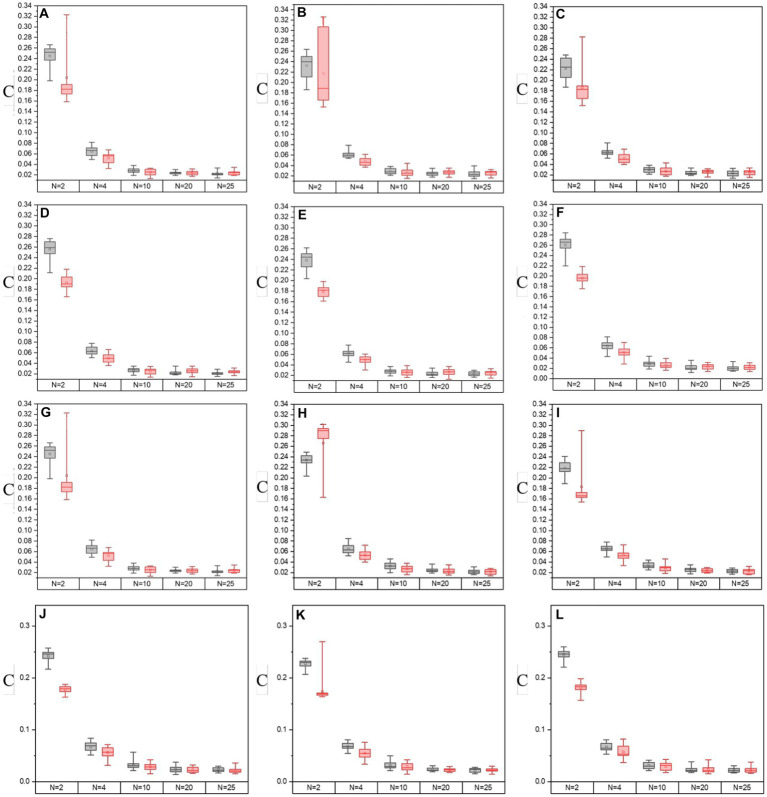
The values of C of groups with varying segmentation modes from I-S-P3, I-S-P4 of off-diagonal MMEs. **(A–F)** m12, m13, m21, m23, m31, and m32 for glioma region, **(G–L)** m12, m13, m21, m23, m31, and m32 for the non-glioma region.

**Figure 9:**
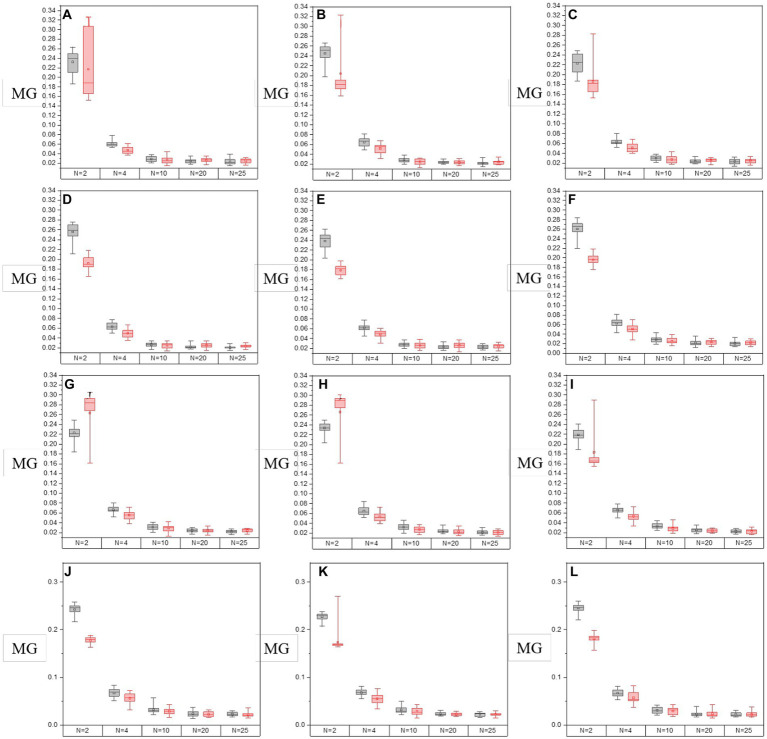
The values of MG of groups with varying segmentation modes from I-S-P3, I-S-P4 of off-diagonal MMEs. **(A–F)** m12, m13, m21, m23, m31, and m32 for glioma region, **(G–L)** m12, m13, m21, m23, m31, and m32 for the non-glioma region.

In Figures 8, 9, it shows the results of the evaluation of C and MG on enhanced images with different segmentation modes when the enhancement processes are performed on all off-diagonal MMEs in glioma and non-glioma regions, based on multi-sampled tests. The gray bars refer to the results from P3-enhanced images, while the red bars refer to the results from P4-enhanced images. Specifically, it shows the evaluation results of C tested on the off-diagonal MMEs of glioma and non-glioma regions in Figure 8, and in Figure 9 it shows the results of the assessment coefficient MG.

Based on the results of assessment coefficients in Figures 8, 9, it can be seen that the C and MG values of P3 and P4 enhanced images with *N* = 2 for both glioma and non-glioma regions are higher than other segmentation modes, indicating that enhanced images at *N* = 2 perform better. In addition, it seems that P3-enhanced images perform better than P4-enhanced images with different segmentation modes. However, in combination with the results of the visual effect from Figure 7, the tradeoff between small and large-segmenting modes should be considered. Technically, a smaller N usually results in miscalculation of the P3 and P4 enhancement matrices, and it is difficult to add statistical information to the enhanced images, while a larger N lacks the specificity of P3 and P4 enhancement matrices, which is caused by the inclusion of both glioma and non-glioma information for some sub-regions, so the enhanced images at *N* = 2, 4, and 10 are more practically useful for the display of enhanced PIT images to show the visual effect and to consider the results of evaluation coefficients.

**Figure 10:**
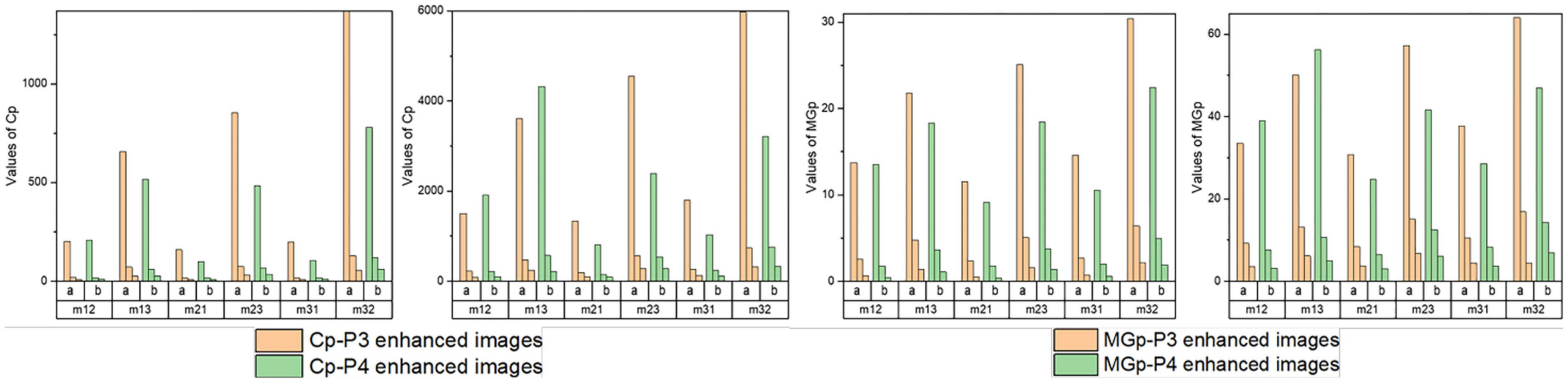
Normalized assessment coefficients of enhanced images with *α* = 0.9, *β* = 0.1 and *N* = 2, 4, 10 of off-diagonal MMEs. **(A)** Cp of glioma regions, **(B)** Cp of non-glioma regions, **(C)** MGp of glioma regions, **(D)** MGp of non-glioma regions.

Then, the normalized assessment coefficients Cp and MGp of the off-diagonal MMEs with *N* = 2, 4, and 10 of P3 and P4 enhanced images with the optimal overlapping scale *α* = 0.9 and *β* = 0.1 are tested and shown in Figure 10. Similarly, group (a) represents Cp or MG*p* values of the P3 enhanced images, while group (b) represents the Cp or MGp values of the P4 enhanced images and the three bars for each group refer to the values of enhanced images with *N* = 2, 4, and 10. From Figure 10, firstly, it had the best results with *N* = 2 for the highest promotion Cp and MGp, then *N* = 4, with *N* = 10 the worst. However, it gained enhancement effect when *N* = 10 according to the Figures 4C–F from section 3.4, and it is preferable and practically useful for the clinical or surgical application when *N* = 4, combined with the results of the visual effect of enhanced images shown in Figure 7. Secondly, it can be seen that both Cp and MGp obtained better-enhanced results for non-glioma regions. Besides, it is obvious that P3-enhanced images have larger values of Cp and MGp, compared with that of P4-enhanced images. In addition, m23 and m32 gained higher Cp and MGp for both glioma and non-glioma regions, indicating that the enhancement of them could lead to better results and may provide more useful clinical information in further studies.

Based on multi-sample testing, it appears that P3-enhanced images with an overlapping scale of *α* = 0.9 and *β* = 0.1 at *N* = 4 yielded the best enhancement results. In addition, the procedure for testing significance in *t*-test statistics for P3-enhanced images uses an overlap scale of *α* = 0.9 and *β* = 0.1 of *N* = 4, and the original images were examined to determine the stability of the enhancement results for parameter C and MG for glioma and non-glioma regions, which showed a statistically significant difference (*p* < 0.05 is significant). The results are presented in Tables 4, 5.

**Table 4:** The *p* values between P3 enhanced images using overlay scale of *α* = 0.9 and *β* = 0.1 with *N* = 4 and the origin images of the off-diagonal MME for parameter C and MG of glioma regions.

*P* values for parameter C						……
m12	m13	m21	m23	m31	m32	……
1.14049e^−20^	3.18e^−20^	6.4471e^−22^	5.41e^−23^	5.6179e^−22^	8.67e^−18^	……
……	*P* values for parameter MG					
……	m12	m13	m21	m23	m31	m32
……	2.789e^−26^	1.14e^−26^	4.677e^−28^	1.89e^−28^	8.603e^−27^	1.97e^−24^

**Table 5:** The *p* values between P3 enhanced images using overlay scale of *α* = 0.9 and *β* = 0.1 with *N* = 4 and the origin images of the off-diagonal MME for parameter C and MG of nonglioma regions.

*P* values for parameter C						……
m12	m13	m21	m23	m31	m32	……
2.154e^−23^	5.38e^−19^	3.513e^−24^	2.45e^−19^	4.146e^−23^	4.07e^−21^	……
*P* values for parameter MG						
m12	m13	m21	m23	m31	m32	……
2.239e^−30^	1.74e^−28^	6.656e^−31^	1.98e^−28^	1.016e^−30^	2.22e^−29^	……

## 4. Discussion

Based on the multi-sample tests, the proposed PIT image enhancement method is valid for both diagonal and off-diagonal MMEs, especially for off-diagonal MMEs, and assessment coefficients are calculated to quantify the results of enhanced PIT images. In addition, the studies also test and determine the overlapping scale α and β between the original PIT images and enhancement images and segmenting modes N of original images that affect the enhancement effect. However, more detailed and systematic tests remain to improve the display of enhanced images and evaluate the enhancement results. Future work consists of visualizing these enhanced PIT images which can be programmed in near real-time into a small 3 × 3 Mueller matrix polarization experimental system for the identification of gliomas and glioma residues in an intraoperative environment.

## 5. Conclusion

In this article, a PIT image enhancement method was proposed based on the calculations of P3 and P4, and then the PIT images and the assessment coefficients C, Cp, MG, and MGp were used to evaluate the effect of enhancement with this method, and it has been proved to be practically effective and useful when applied to the images of MMEs, especially off-diagonal MMEs, based on multi-sample tests. The research also proposed and tested two important factors affecting the enhancement effect, namely the overlapping scale between the original image and enhancement matrices and the segmentation mode of the original images. Based on multi-sample testing, it appears that the P3-enhanced images with an overlapping scale of *α* = 0.9 and *β* = 0.1 with *N* = 4 yielded the best enhancement results. This PIT image enhancement method can greatly improve the contrast and detailed texture information of MMEs images, which can provide more useful clinical information, and can be used as a basis for calculating PIT parameters for identifying the glioma from non-glioma regions, which lays a foundation for further application to identify gliomas and residues in the intraoperative environment with PIT.

## Data availability statement

The original contributions presented in the study are included in the article/supplementary material, further inquiries can be directed to the corresponding author.

## Author contributions

Y-RL carried out experiment, performed the data analysis, and responsible for the writing of the manuscript. C-FL guided the interpretation of polarization characteristics of glioma samples and supporting the deployment of clinical resources. H-QZ and YM-O helped to collect the data and participated in the experiments. JW supervised the work. All authors contributed to revising the manuscript and approved the final version.
